# Supplementary material for: A Single-Chain Mpox mRNA Vaccine Elicits Protective Immune Response in Mice
Source: Vaccines (Basel). 2025 May 13;13(5):514. doi: 10.3390/vaccines13050514 (PMC12115521; doi:10.3390/vaccines13050514)
Supplement: Supplementary file 1 [file vaccines-13-00514-s001.zip › vaccines-3600684-supplementary material.pdf]

A single-chain mpox mRNA vaccine elicits protective immune response in mice

Supplementary Materials

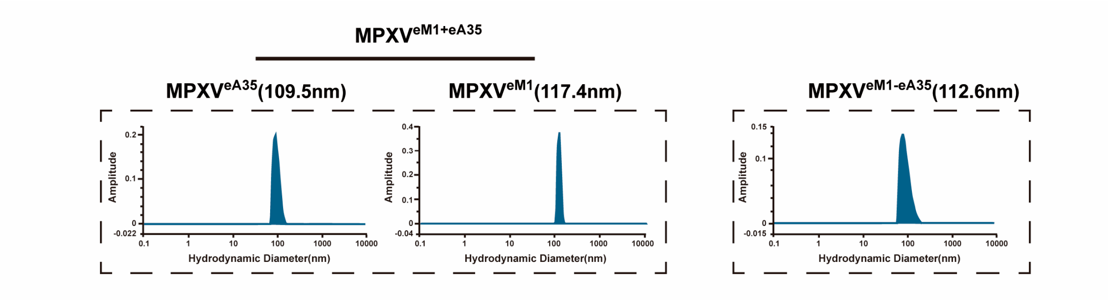

Supplemental figure S1 Representative Size Distribution Profiles of MPXV^{eA35}, MPXV^{eM1}, and MPXV $^{eM1-eA35}$.

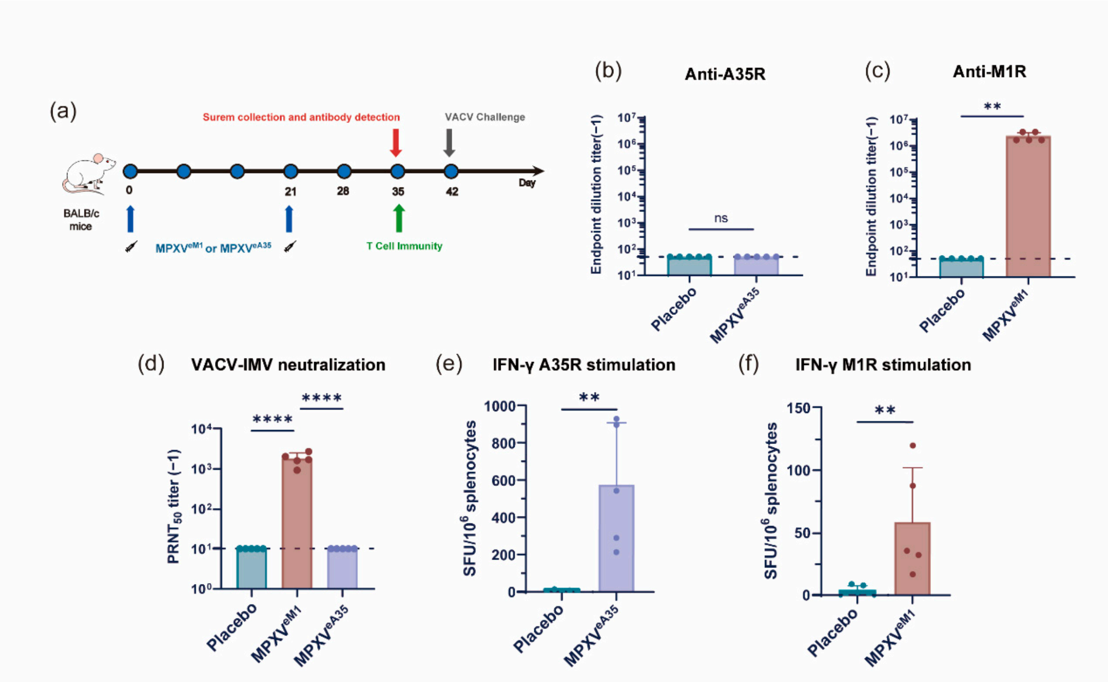

Supplemental figure S2 Evaluation of immunogenicity and protective efficacy of single antigen vaccines. **(a) Immunization Schedule for MPXV^{eA35} and MPXV^{eM1}.** Female BALB/c mice were intramuscularly injected with MPXV^{eA35}, MPXV^{eM1} or placebo, followed by a booster with the same dose three weeks later. Serum and splenocyte samples were collected at specified time points. **(b-c) ELISA Measurement**

of A35R- and M1R-Specific IgG Antibody Titers. (d) PRNT Measurement of Neutralizing Antibody Levels Against VACV in Mouse Serum. (e-f) ELISpot Assessment of Cellular Immune Responses Induced by single antigen vaccines. Splenocytes from BALB/c mice were harvested on day 14 post the final immunization and stimulated with peptide pools of A35R (e) and M1R (f) to measure the secretion of cytokines IFN-γ (e, f). Data are presented as mean ± SEM. Statistical significance was analyzed using the Mann-Whitney test or one-way ANOVA with multiple comparison tests (ns, not significant, **p < 0.01, ***p < 0.001, ****p < 0.0001).

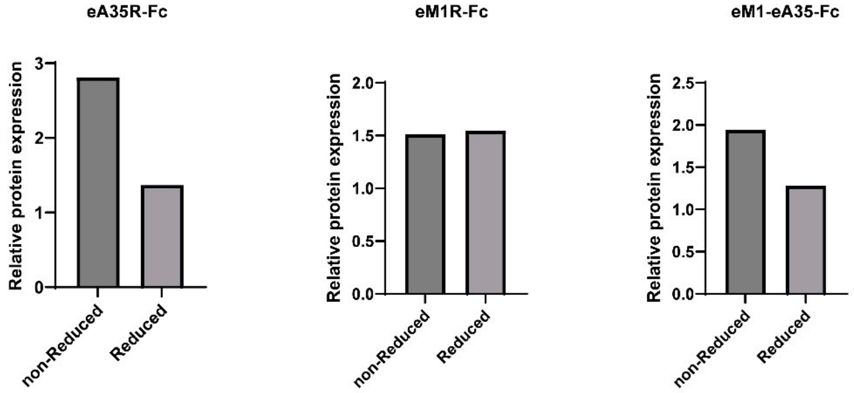

Supplemental figure S3 Integrated density (IntDen) values of eA35R-Fc, eM1R-Fc, eM1-eA35-Fc normalized to GAPDH.

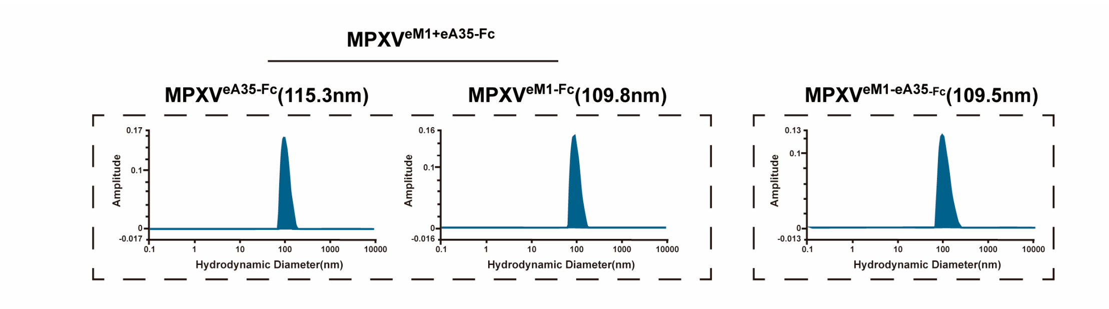

Supplemental figure S4 Representative Size Distribution Profiles of MPXV$^{eA35\text{-}Fc}$, MPXV$^{eM1\text{-}Fc}$, and MPXV $^{eM1\text{-}eA35\text{-}Fc}$.
